# Transition Metal Selenide-Based Anodes for Advanced Sodium-Ion Batteries: Electronic Structure Manipulation and Heterojunction Construction Aspect

**DOI:** 10.3390/molecules29133083

**Published:** 2024-06-28

**Authors:** Lingxiao Li, Shuotong Wang, Jinyang Peng, Junliang Lai, Heng Zhang, Jun Yang

**Affiliations:** School of Material Science and Engineering, Jiangsu University of Science and Technology, Zhenjiang 212003, China; lix.poon@foxmail.com (L.L.); 15918698019@163.com (S.W.); 13350841933@163.com (J.P.); e1150882@163.com (J.L.); hengzhang@just.edu.cn (H.Z.)

**Keywords:** transition metal selenides, anode, sodium-ion batteries, electronic modulation, heterojunction construction

## Abstract

In recent years, sodium-ion batteries (SIBs) have gained a foothold in specific applications related to lithium-ion batteries, thanks to continuous breakthroughs and innovations in materials by researchers. Commercial graphite anodes suffer from small interlayer spacing (0.334 nm), limited specific capacity (200 mAh g^−1^), and low discharge voltage (<0.1 V), making them inefficient for high-performance operation in SIBs. Hence, the current research focus is on seeking negative electrode materials that are compatible with the operation of SIBs. Many studies have been reported on the modification of transition metal selenides as anodes in SIBs, mainly targeting the issue of poor cycling life attributed to the volume expansion of the material during sodium-ion extraction and insertion processes. However, the intrinsic electronic structure of transition metal selenides also influences electron transport and sodium-ion diffusion. Therefore, modulating their electronic structure can fundamentally improve the electron affinity of transition metal selenides, thereby enhancing their rate performance in SIBs. This work provides a comprehensive review of recent strategies focusing on the modulation of electronic structures and the construction of heterogeneous structures for transition metal selenides. These strategies effectively enhance their performance metrics as electrodes in SIBs, including fast charging, stability, and first-cycle coulombic efficiency, thereby facilitating the development of high-performance SIBs.

## 1. Introduction

In view of the recent rapid development of the international economy and the energy consumption ratios, it is important to investigate and use important additional energy mixes. Lithium-ion batteries (LIBs) are, today, the most important energy technology in the world [1,2,3]. However, the development of LIBs energy technology is severely limited due to lithium’s deficiency and abnormal distribution and high production costs, which makes it difficult to meet future large-scale energy demands [4,5,6]. In contrast to LIBs, sodium-ion batteries (SIBs) have advantages such as abundant and easily accessible raw material reserves, high abundance, good rate performance, good performance at high and low temperatures, and lower prices [7,8]. They can provide long-term supply guarantees for the consumption needs of modern society.

However, sodium ions have a larger radius than lithium ions, resulting in lower capacity and making them more prone to damaging the physical structure of the active material during insertion/extraction, leading to a decrease in material cycling stability, which fails to meet people’s needs [9]. Since the function of SIBs depends mainly on the function of electrical materials, people need to explore novel anode materials to replace traditional graphite anodes [10,11]. In recent years, designing and synthesizing negative electrode materials with high reversible capacity and stability has become a research hotspot [12]. Hard carbon and Ge are often used as the negative electrode materials of SIBs. Hard carbon can be prepared from cheap carbon sources with abundant raw materials and low cost, and the volume of hard carbon changes little during the charge and discharge processes, which reduces the risk of internal short circuit and other safety hazards of the battery. However, the low capacity of hard carbon makes the energy that SIBs can store low. Ge, as a negative electrode material, has a high theoretical specific capacity and fast electronic conductivity, but its volume expansion during charge and discharge makes it difficult to avoid the problem of poor stability, and it is not the most preferred negative electrode material for SIBs. Recent studies on advanced negative electrode materials have shown that transition metal selenides (TMSs), transition metal oxides (TMOs), and transition metal sulfides (TMDs) are promising choices [13,14,15,16]. TMOs and TMDs have become potential candidates for SIB negative electrodes due to their high theoretical power and low cost. But their lower conductivity, poor cycle stability, and environmental and safety issues limit their use. TMSs and TMOs, like TMDs, exhibit many similar characteristics. As electrodes for SIBs, they all have similar conversion-based charging and discharging mechanisms.

TMSs exhibit some unique characteristics compared to TMOs and TMDs. Firstly, due to the high density of selenium, the volumetric energy density of metal selenides is much higher than that of metal oxides or sulfides. Subsequently, the conductivity of Se is higher than that of O and S; thus, TMSs have a higher electronic conductivity, which is beneficial for improving the multiplicity of electrode materials. Secondly, selenides serve as a viable alternative to other anode materials (e.g., germanium) and hard-carbon negative electrodes, as they can suppress the formation of dendritic crystals in certain battery systems, thereby enhancing battery safety and lifespan, and reducing the risk of short circuits and internal damage. Concurrently, the ordered nanoarray configuration of TMSs offers a solution to the limitations of conventional electrode fabrication methods [17,18]. It achieves this by reducing ion diffusion distances, enhancing the interface between electrode materials and electrolytes, preventing electrode material aggregation during faradaic reactions, and boosting the loading of active materials. Additionally, the inherent metallic properties of multicomponent selenides, synergistic effects among multiple metal ions, superhydrophilicity of material surfaces, unique honeycomb arrays, and robust multi-structured nature all contribute to their array of advantages. Moreover, compared to TMOs and sulfides, the M-Se bonds in TMSs are weaker, which favors conversion reactions kinetically [19,20]. Additionally, their unique layered structure facilitates ion diffusion. These advantages contribute to the good cycling stability of metal selenides. Therefore, selenide materials demonstrate better electrochemical performance in SIBs, enhancing overall battery efficiency and stability.

In the field of TMSs, the research focus is primarily on iron-based selenides, cobalt-based selenides, nickel-based selenides, and molybdenum-based selenides [21,22]. These selenides have attracted considerable interest because of their distinct properties and characteristics. This paper primarily focuses on iron-based selenides, cobalt-based selenides, nickel-based selenides, and molybdenum-based selenides, aiming to improve the performance of TMSs in SIBs from two perspectives: heteroatom doping and heterojunction construction. The goal is to enhance their cycling lifespan and energy storage capacity.

**Figure 1:**
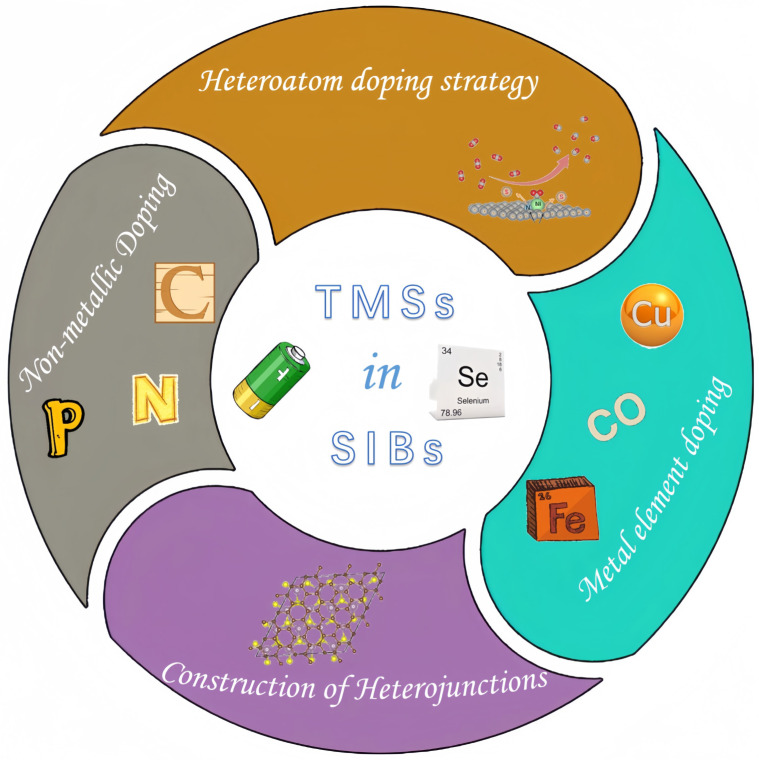
Diagram of the regulation of transition metal selenides.

This paper focuses on the application of TMSs in SIBs, primarily exploring their impact on SIBs from two perspectives: heteroatom doping and the construction of heterojunctions. An overview can be seen in Figure 1.

## 2. Transition Metal Selenides as Electrode Materials

### 2.1. Transition Metal Selenides

**Figure 2:**
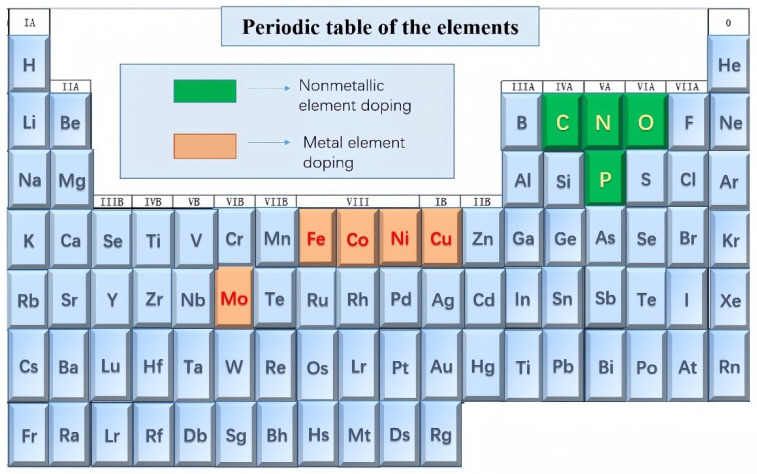
Transition metal selenides and their doping elements.

In recent years, metal selenides have received increasingly widespread attention, with a significant increase in the number of electrode products based on metal selenides. This paper primarily focuses on doping and heterojunction construction using the elements shown in Figure 2. The elements highlighted in darker brown represent metallic element doping, while those highlighted in darker green represent non-metallic element doping. This comprehensive review article covering the latest advancements is anticipated to offer valuable and compelling insights to bolster future research endeavors concerning metal selenides, particularly in the realms of energy storage/conversion and other related applications.

#### 2.1.1. Common Transition Metal Selenides Anodes and Their Properties

Among them, iron-based selenides (FeSe_x_) are mainly divided into FeSe_2_, FeSe, and Fe_7_Se_8_. Their corresponding theoretical capacities in SIBs are 501.5, 397.6, and 419.3 mA h g^−1^, respectively [16]. Here, we delve into the physical and chemical properties of FeSe_2_. Firstly, the structure of FeSe_2_ belongs to the orthorhombic crystal system, comprising FeSe_4_ tetrahedra and Se_2_ dimers. It has a small bandgap of only 1.0 eV, and the lattice distance of the (111) plane is approximately 0.2573 nm, with a potential barrier height of about 0.371 eV [23]. For example, Santoshkumar et al. demonstrated the growth of FeSe_2_ layered nanoparticles consisting of irregular nanoparticles and cavities using a KCl modeled hot-solvent method [24]. This rough, sheet-like nanostructure ensures good adhesion to the substrate, and the cavity ensures greater porosity. Therefore, the electrode material has better cycle stability (95%) and higher capacitance (524 mA h g^−1^). This highlights the potential application of FeSe_2_ in energy storage, with an excellent cycling life and efficient energy storage performance.

Within this category, cobalt-based selenides (CoSe_x_) are primarily categorized into CoSe_2_, Co_0.85_Se, Co_3_Se_4_, CoSe, and Co_9_Se_8_. These variants possess respective theoretical capacities in SIBs of 494.5, 441, 435, 388.7, and 369 mA h g^−1^, accordingly [25]. Below, we will first introduce the physical and chemical properties of CoSe. CoSe has a hexagonal crystal structure, forming a three-dimensional network of octahedra. This unique structure provides it with excellent sodium-ion migration and electrochemical stability. It has a relatively small bandgap of only 1.53 eV, with an interlayer distance of 1 nm. The conductivity typically ranges from 10^−3^ to 10^2^ S cm^−1^; however, low conductivity may result in high resistance and poor rate performance. Charge population analysis of CoSe reveals its peculiarity, with Co atoms carrying positive charges (+0.36 eV) and Se atoms carrying negative charges (−0.36 eV). When the current density is 0.1 A g^−1^, CoSe can provide reversible capacitance up to 435 mA h g^−1^; even after 500 cycles at the voltage density of 2 A g^−1^, it remains at 282 mA h g^−1^ [26]. After 4000 cycles with a current density of 5 A g^−1^, the capacity remains at 313 mA h g^−1^, and even with high current density of 20 A g^−1^, the reversible capacitance remains at 175 mA h g^−1^. The aforementioned properties highlight the promising application potential of CoSe in areas such as SIBs, offering robust backing for its utilization in advanced energy storage systems. CoSe_2_, belonging to the same family of metal selenides, exhibits remarkable physical and chemical properties. The typical hexagonal crystal structure determines its unique electrical and structural characteristics. CoSe_2_ has a relatively small bandgap, typically ranging from 1.0 to 2.0 eV, while its conductivity values lie within a wide range, generally between 10^−3^ and 10^2^ S cm^−1^. Detailed analysis of the crystal structure reveals characteristic dimensions of key crystal planes, such as the interplanar spacing of the (200) plane at 0.293 nm, the lattice distance of the (221) plane at 0.1956 nm, and the (111) plane at 3.383 nm. These crystal plane parameters are crucial for explaining the performance of CoSe_2_ in electronic and ionic transport. In SIBs, CoSe_2_ exhibits outstanding performance as an electrode. When cycled continuously at a current density of 1.0 A g^−1^ for 1800 cycles, it maintains excellent performance, with a capacity of 410 mA h g^−1^ [27]. Throughout 1800 cycles of charging and discharging within a voltage span of 0.5–3.0 V, CoSe_2_ maintains a notable discharge capacity of 410 mA h g^−1^ when operated at a rate of 1 A g^−1^. More notably, at extremely high current densities (10 and 50 A g^−1^), CoSe_2_ demonstrates an unprecedented rate performance, achieving capacities of 354 mA h g^−1^ and 97 mA h g^−1^, respectively. These results indicate the enormous application potential of CoSe_2_ in the field of batteries, providing new directions for the development of high-performance energy storage systems.

Nickel-based selenides (NiSe_x_) are primarily divided into NiSe_2_, NiSe, and Ni_0.89_Se, with theoretical capacities in SIBs of 494.8, 389.4, and 416 mA h g^−1^, respectively [28,29]. We focus on NiSe_2_, a member of the nickel-based selenide family, which typically exhibits a hexagonal crystal structure, where Ni and Se elements construct a three-dimensional network. NiSe_2_ has a bandwidth of about 1.47 eV and has impressive electrochemical properties [30]. Initially, it showed a significant discharge capacity of 755 mA h g^−1^, although after 100 cycles, the discharge capacity of 35 mA h g^−1^ decreased at a rate of 0.2 A g^−1^ [31]. With a high current density of 5 A g^−1^, the initial discharge and charging capacity are 478 mA h g^−1^ or 469 mA h g^−1^. NiSe_2_ nano-octahedra demonstrate remarkable rate capabilities, delivering capacities of 472, 360, and 175 mA h g^−1^ at rates of 1, 2, and 20 A g^−1^, respectively [32]. More notably, the NiSe_2_ anode demonstrates an outstanding cycle life, maintaining 4000 cycles at a current density of 5 A g^−1^ and retaining a high capacity of 313 mA h g^−1^. These results emphasize the potential application of NiSe_2_ in the field of energy storage, particularly its outstanding performance in high rate capability and long cycle life. Another remarkable nickel-based selenide is Ni_0.85_Se, which exhibits a honeycomb-like nano-sheet array structure. This unique honeycomb structure imparts intrinsic metallic properties and superhydrophilicity, further enhancing its attractiveness for various applications. Ni_0.85_Se demonstrates excellent cycling stability, with a capacitance retention rate as high as 90.1% after 5000 cycles, while also exhibiting outstanding specific capacitance [33]. Its bandgap is approximately 1.75 eV, further indicating its potential utility in the field of electrochemistry.

Molybdenum-based selenides mainly refer to MoSe_2_, which typically exhibits a layered structure of Se-Mo-Se, with interlayer distances ranging from 0.64 nm to 0.65 nm, formed due to weak van der Waals forces. MoSe_2_ features a theoretical capacity of up to 422 mA h g^−1^ in SIBs, along with the advantages of low cost and environmental friendliness. It has a bandgap of approximately 1.1 eV and shows direct transport characteristics under pressure, gradually narrowing the bandgap and eventually leading to metalization [34,35]. However, due to its low electrical conductivity, it is prone to increased impedance, affecting its rate performance. During sodium storage, MoSe_2_ generates polyselenide intermediates, leading to side reactions and increased polarization, resulting in the inability to fully convert its theoretical capacity into reversible capacity. The enlarged interlayer spacing on the MoSe_2_ (002) crystal surface is 0.664 nm, while MoSe_2_ nanoflower electrodes still exhibit a high capacitance retention rate of 90% after 10,000 cycles [36]. Additionally, MoSe_2_ electrode materials without binders have excellent specific capacities of up to 548 mA g^−1^ [37]. These properties highlight the potential application of MoSe_2_ in the field of energy storage and provide research directions for further optimization in battery technology [38].

#### 2.1.2. Issues of Transition Metal Selenide Anodes

The charge–discharge mechanism of selenides primarily involves multi-step reactions and phase transformations. First, during the charging process, the bonds between the transition metal and selenium (Se) in the selenide gradually break, releasing selenium atoms. These selenium atoms further combine with sodium ions (Na⁺) to form sodium selenides (e.g., Na_2_Se). Meanwhile, the transition metal undergoes redox reactions, changing its oxidation state. Conversely, during the discharge process, these reactions occur in reverse. The sodium selenide compounds decompose, releasing sodium ions and selenium atoms, which recombine with the transition metal to restore the original selenide structure. However, transition metal selenides (TMSs) still have some shortcomings. Metal sulfides have lattice defects, which can reduce electron transport rates [39,40]. Additionally, the crystal structure of metal selenides may also affect the free movement of electrons. Complex or highly distorted crystal structures may restrict electron flow within the lattice. Moreover, if metal selenide samples contain local defects or non-uniformities, the current may be affected by these regions. These factors contribute to the inadequate conductivity of transition metal selenides, leading to increased resistance and decreased power density. In addition to the conductivity issues, when used as the cathode material in SIBs, metal selenides often experience volume expansion due to changes in their crystal structure caused by sodium-ion insertion and extraction during charge and discharge cycles. This results in a decrease in the effective electrode capacity, affecting the battery’s cycle life and performance. Apart from these two defects, TMSs also suffer from limitations in rate performance, affecting their efficiency in applications requiring rapid charge and discharge [41,42]. They may exhibit poor cycling stability after long-term cycling, have high production costs, making large-scale production difficult, and some TMSs may be susceptible to oxidation or other forms of corrosion in the environment.

Researchers have proposed many solutions to address the aforementioned issues, among which a common approach is to combine metal selenides with carbon materials, such as MXene, rGO, and so on [43]. Shi et al. successfully anchored ZnSe nanoparticles (NPs) firmly onto graphene while simultaneously externally modifying MoSe_2_ nanosheets, ultimately constructing a ZnSe⊂N-C@MoSe_2_/rGO sandwich-layered structure [44]. Using a carbon framework as the support structure for ZnSe and MoSe_2_ helps alleviate their volume expansion and aggregation during charge–discharge cycles. This scaffold has abundant channels and good conductivity, facilitating electrolyte penetration to ensure rapid charge and ion transfer throughout the electrode, thereby reducing unnecessary wastage of active materials. Additionally, the porous structure aids in storing a large amount of Na^+^, resulting in a capacity of 319.4 mA h g^−1^ after 1800 cycles at 1 A g^−1^ and 177.7 mA h g^−1^ after 5000 cycles at 10 A g^−1^, demonstrating excellent cycling performance under high rate cycling.

Cu_1.75_Se-MXene-CNRib, when the Na^+^ diffusion coefficient is in the range of 10^−8^ to 10^−9^ cm^2^ s^−1^, exhibits a maximum reversible Na^+^ storage capacity of 536.3 mA h g^−1^ at 0.1 A g^−1^ [45]. After 400 cycles, it still maintains a stable K^+^ capacity of 305.6 mA h g^−1^ at a current density of 1.0 A g^−1^. However, these materials, combining metal chalcogenides with MXene, rGO, and others, still have some drawbacks. One of the challenges lies in ensuring the interface consistency and effective bonding between transition metal chalcogenides and MXene and rGO. Since carbon has a relatively low theoretical capacity in SIBs, the combination of metal chalcogenides with MXene and rGO may lead to a decrease in the theoretical capacity in SIBs. Additionally, there may be differences in the conductivity between metal chalcogenides and MXene and rGO, resulting in localized resistance during electron transport, which could negatively impact applications sensitive to electron transport. Moreover, the synthesis process of metal chalcogenides and MXene and rGO composite materials is complex and requires high preparation costs, making it difficult to achieve large-scale production and application. Furthermore, this composite material may experience performance degradation during long-term cyclic stability testing in electrochemical applications, a problem that still needs to be addressed.

## 3. Modification of Transition Metal Selenide Electrode Materials

To address this, researchers have begun to investigate alternative methods to enhance the theoretical capacity of TMSs in SIBs. Two approaches have been proposed: heteroatom doping and constructing heterogeneous structures. The following will introduce these two methods.

### 3.1. Heteroatom Doping Strategy

Types of dopants with impurity atoms can generally be classified into non-metallic element doping and metallic element doping. Typically, impurity atoms can be introduced by two methods: using precursor materials rich in impurity atoms or directly adding reactive impurity atom sources to the reaction process. Since the introduced impurity atoms possess significantly different physicochemical properties from the host atoms, they form unique electron environments and bonding interactions around the substitution positions. Considering that the majority of electrode materials exhibit semiconductor properties, the introduction of impurity atoms with varying valence states serves to enhance the concentration of either electrons (in n-type semiconductors) or holes (in p-type semiconductors). In n-type semiconductors, the additional electrons can transition to the conduction band, thus increasing the population of free electrons. Conversely, in p-type semiconductors, the heightened number of holes lowers the energy barrier for electrons to move from the valence band to the conduction band. Impurity atom doping is a convenient and effective method that increases the active sites of materials by changing the lattice structure, increasing dislocations, etc., thereby improving conductivity. Additionally, the energy levels occupied by the charge carriers induced by impurity atoms are called impurity levels, which have the effect of narrowing the semiconductor bandgap. All these effects can alter the electron state and electron structure, thereby enhancing electron conductivity and, subsequently, improving the rate performance of electrode materials. In recent years, this strategy has been widely used to improve the negative electrode materials of LIBs. Common non-metallic element doping includes N, S, P, F, and B doping, while common metallic element doping includes Cu, Cr, Ti, etc. [46,47].

#### 3.1.1. Non-Metallic Doping

Non-metallic doping can enhance the electrochemical performance of materials. Doing so will result in enhanced active sites, thus improving the energy storage capabilities of electrode materials. It can also enhance the structural stability of electrode materials, improve material cyclic stability, and prolong the lifespan of electrode materials. Through non-metallic doping, reaction kinetics can be improved, making electrode materials suitable for high-power applications, thereby enhancing battery performance [48]. Furthermore, non-metallic doping may be more cost-effective compared to metallic doping, making it economically viable. Below, we will first introduce examples of non-metallic doping. Ge et al. prepared nitrogen-doped, carbon-encapsulated FeSe_2_ nanorods (FeSe_2_/N-C) [49]. These intelligently structured nanorods with uniform carbon coating exhibited ultra-long cycling stability at high rates. For instance, even after 10,000 cycles at a high rate of 10 A g^−1^, they still retained a reversible capacity of 308 mA h g^−1^. Doping slightly reduced the bandgap of MoSe_2_ and enhanced its conductivity. Currently, there are numerous efforts focusing on utilizing MoSe_2−x_S_x_ as an intermediate composite material derived from these two sister molybdenum disulfides. Yang et al. synthesized a composite material consisting of MOF-derived CNTs, nitrogen-doped carbon, and CoSe_2_ via a solvothermal-assisted selenization process (CNT/CoSe_2_@NC) [50]. TEM analysis validated the necklace-like morphology observed in the CNTs/CoSe_2_@NC composite material. The incorporation of CNTs improved the cycling performance of the CoSe_2_@NC composite material. Benefiting from the mechanical reinforcement offered by CNTs, the CNT/CoSe_2_@NC composite material exhibited a capacity retention of 80% after 120 cycles, surpassing that of CoSe_2_@NC (15.2%). In another study, Wang and colleagues meticulously analyzed the electrochemical characteristics of composite materials consisting of Na_2_S and Na_2_Se using a combination of experimental investigations and density functional theory (DFT) computations [51]. Among the three interfaces (Na_2_O/Na_2_S, Na_2_O/Na_2_Se, and Na_2_S/Na_2_Se), DFT calculations revealed that the diffusion barrier at the Na_2_S/Na_2_Se interface was the lowest, at 0.39 eV. Zhang et al. employed the cobalt organic framework (ZIF-67) as a sacrificial template to investigate the formation of CoSe/C mesoporous dodecahedra under varying temperatures within the original nitrogen-doped carbon matrix, alongside Se powder [52]. The small size of CoSe nanoparticles, approximately 15 nm, enabled their entrapment within the mesoporous carbon framework. CoSe/C composite material is a new negative SIBs electrode material with significant power and performance. The specific capacities of the composite electrode were 597.2 mA h g^−1^, at 0.2 A g^−1^, and 361.9 mA h g^−1^, at 16 A g^−1^, respectively. The CoSe/C dodecahedral structure, used as an SIBs electrode material, showed excellent speed and maintained good cycle stability.

**Figure 3:**
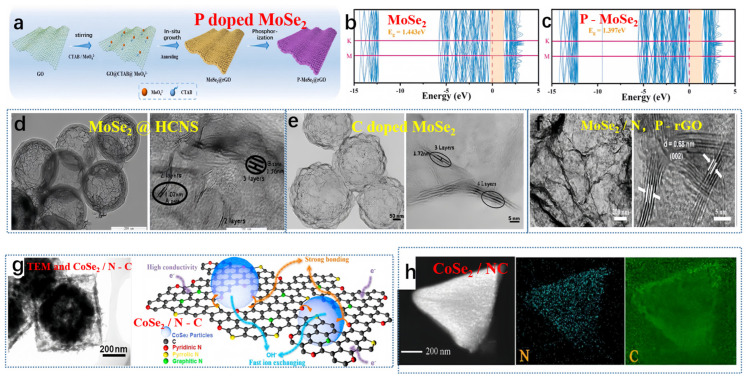
(**a**) The preparation steps of P-MoSe_2_@rGO, (**b**) MoSe_2_ band structures, and (**c**) P-MoSe_2_ band structures. (**a**–**c**) Reproduced from [53] Copyright 2023, Elsevier. (**d**) TEM and HRTEM images of MoSe_2_@HCNS, reproduced from [54] Copyright 2018, John Wiley and Sons. (**e**) TEM and HRTEM images of MoSe_2_-C, reproduced from [55] Copyright 2018, Elsevier. (**f**) TEM and HRTEM images of MoSe_2_/N, P-rGO composite material, reproduced from [56] Copyright 2017, John Wiley and Sons. (**g**) TEM and structure characterization of CoSe_2_/N-C, reproduced from [57] Copyright 2017, Elsevier. (**h**) EDS surface scan of CoSe_2_/NC composite material, reproduced from [58] Copyright 2020, American Chemical Society.

Guo et al. produced P-MoSe_2_@rGO via a straightforward synthesis process, outlined in Figure 3a [53]. The hybridization between Mo-4d and P-3p orbitals caused a shift in the valence band edge toward the Fermi level. Based on the band structure diagram (Figure 3b,c), the direct bandgap of MoSe_2_ measured 1.443 eV, while that of the P-MoSe_2_ system was slightly reduced to 1.397 eV, aligning with the results obtained from the projected density of states (PDOS). Therefore, DFT calculations suggested that P doping effectively enhanced the conductivity of MoSe_2_, accelerating ion/electron transport. Not long ago, Liu and collaborators crafted a high-performing hybrid material, known as MoSe_2_@HCNS. This innovative material incorporates expanded MoSe_2_ nanosheets enclosed within hollow carbon nanospheres, achieved through a simple solution and hydrothermal method (depicted in Figure 3d) [54]. Moreover, MoSe_2_@HCNS exhibited an increased interlayer spacing of the (002) plane, expanding to 1.02 nm (in contrast to pure MoSe_2_: 0.64 nm). Conversely, Liu et al. engineered a yolk-shell architecture of nitrogen-doped carbon (MoSe_2_-C) surrounding MoSe_2_ flakes (illustrated in Figure 3e) [55]. In this study, the interlayer spacing of the (002) plane in MoSe_2_-C was measured at 1.15 nm. Niu et al. reported the growth of MoSe_2_ composite materials on Co-doped N, P-reduced graphene oxide (MoSe_2_/N, P-rGO) plates with an intermediate layer distance of 0.68 nm (Figure 3f) [56]. Zhang et al. synthesized a MOF-derived N-doped CoSe_2_/C double-shell composite material, featuring an internal cavity, double-shell structure, via a self-template approach [57]. The hollow transmission electron microscopy (TEM) image of the CoSe_2_/C composite material is presented in Figure 3g. The creation of the hollow structure in the composite material stemmed from the variation in diffusion rates between cations and anions. At a current density of 2 A g^−1^, the specific capacitance of the double-shell composite material reached 658 F g^−1^. The outstanding electrochemical performance can be attributed to the formation of pores within the carbon framework, the numerous redox reactions facilitated by cobalt, and the superior conductivity of nitrogen-doped carbon, as depicted in Figure 3g. Miao et al. recently detailed the incorporation of CoSe_2_ particles derived from metal-organic frameworks (MOFs) into nitrogen-doped carbon, confirming the presence of each element through energy-dispersive X-ray (EDX) analysis [58]. As depicted in Figure 3h, this composite material demonstrated outstanding cycling stability, retaining 92% of its capacity after 10,000 cycles [58].

**Figure 4:**
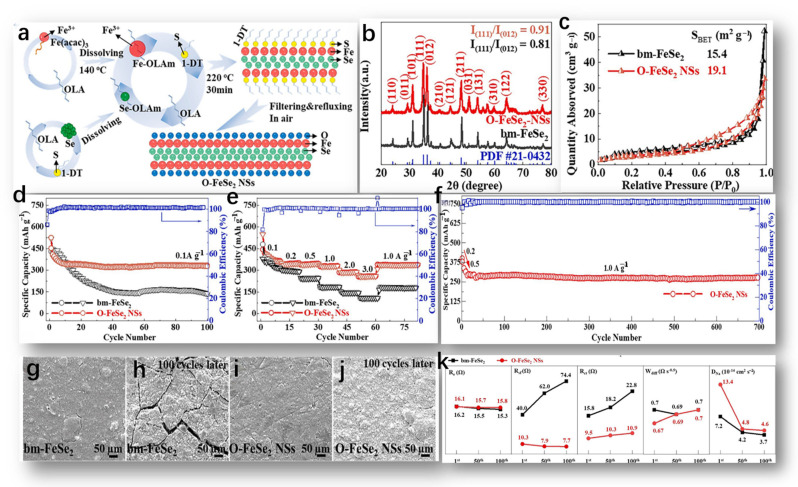
(**a**) The schematic diagram of O-FeSe_2_ NSs preparation, reproduced from [59] Copyright 2015, Elsevier. (**b**) XRD spectra of O-FeSe_2_ NSs and bm-FeSe_2_, and (**c**) nitrogen adsorption/desorption isotherms of bm-FeSe_2_ NSs and O-FeSe_2_ NSs, reproduced from [60] Copyright 2015, John Wiley and Sons. (**d**,**e**) Cycling stability and rate performance of bm-FeSe_2_ and O-FeSe_2_ NSs electrodes, (**f**) long-term cycling performance of O-FeSe_2_ NSs electrode, (**g**–**j**) SEM images of bm-FeSe_2_ and O-FeSe_2_ NSs electrode surfaces before and after 100 cycles at 0.2 A g^−1^, and (**k**) changes in R_e_, R_sf_, R_ct_, W_diff_, and D_Na_ of the two electrodes after different cycle numbers, with all values derived from fitting results. (**d**–**k**) Reproduced from [61] Copyright 2022, Elsevier.

The formation mechanism of O-FeSe_2_ NSs by Huang, S. is depicted in Figure 4a and can be summarized briefly as follows [59]. Firstly, the crystal structure information of O-FeSe_2_ NSs and bm-FeSe_2_ was obtained via XRD. As shown in Figure 4b, the spectra of the two samples correspond to the orthogonal-phase pyrite FeSe_2_ standard graph (JCPDS # 21-0432, spatial group: pmnn), suggesting that FeSe_2_ was successfully synthesized using both methods [60]. The distinct diffraction peaks observed in the XRD spectra affirmed the excellent crystallinity of both samples, and the absence of additional peaks associated with impurities (such as FeO_x_, SeO_x_, FeSe, Fe_3_Se_4_, FeS, etc.) underscored the high purity of both samples. Furthermore, the calculated intensity ratio of crystal planes (111) to (012) in bm-FeSe_2_ was 0.81, lower compared to O-FeSe_2_ NSs at 0.91, suggesting differing crystal growth orientations in the two samples. The nitrogen adsorption/desorption isotherm of the sample is shown in Figure 4c.

The bm-FeSe_2_ curve had typical type IV properties, where the hysteresis loop, H2, indicates the accumulation of nano/microparticles. On the contrary, O-FeSe_2_-NS had a similar adsorption/desorption branch, N_2_, but a hysteresis loop, H4, indicating the presence of pores due to the layer structure. Figure 4d shows the cyclic stability of bm-FeSe_2_ and O-FeSe_2_ nanoplates [61]. Initially, the discharge/charge ratios of bm-FeSe_2_ and O-FeSe_2_ nanochips were 430.0/518.5 mA h g^−1^ and 453.6/525.9 mA h g^−1^, respectively. The initial Coulomb efficacy was 82.9% and 86.2%, respectively. However, bm-FeSe_2_ experienced considerable capacity degradation in subsequent cycles. After only 20 cycles, the capacity of bm-FeSe_2_ decreased significantly compared to O-FeSe_2_ nanosheets. In contrast, the O-FeSe_2_ nanosheets electrode maintained high discharge/charge-specific capacities of 328.0/331.0 mA h g^−1^ and a high CE of 99.1%, even after 100 cycles, demonstrating superior cycling stability and reversibility. Figure 4e compares the multiplication capacity of two electrodes at different current densities (ranging from 0.1 to 3.0 A g^−1^). At current densities of 0.1, 0.2, 0.5, 1.0, 2.0, and 3.0 A g^−1^, the discharge/charge capacities of O-FeSe_2_ nanosheets were approximately 385.4/387.0, 343.9/347.2, 341.3/343.5, 326.8/327.3, 285.8/286.6, and 258.1/258.2 mA h g^−1^, respectively. Of special mention is the fact that, at high current densities of 2.0 and 3.0 A g^−1^, the capacity of O-FeSe_2_ NSs was almost twice that of bm-FeSe_2_. This underscores the beneficial impact of abundant oxygen on both cycling and rate capabilities. In addition, as shown in Figure 4f, the high cyclic performance of O-FeSe_2_ NSs was assessed. Figure 4g–j shows SEM images representing NSs’ surface morphology of bm-FeSe_2_ and O-FeSe_2_ electrodes before and after 100 cycles. In the absence of electrochemical reactions, both electrode surfaces (Figure 4g,i) exhibited similar crack formations and surface roughness. Nevertheless, following 100 cycles, the bm-FeSe_2_ electrode’s surface (illustrated in Figure 4h,j) exhibited pronounced and enlarged cracks, suggesting substantial volume fluctuations throughout repeated discharge and charge cycles. In contrast, the electrode made of O-FeSe_2_ NSs exhibited finer cracks and a more polished surface, implying that the incorporation of a considerable amount of oxygen atoms helps alleviate the expansion of the electrode volume. The fitting outcomes and calculated parameters of R_e_, R_ct_, R_sf_, W_dif_, and D_Na_ are depicted in Figure 4k.

#### 3.1.2. Metal Element Doping

Compared to non-metal doping, metal doping can better enhance the conductivity of materials because it introduces additional electron conduction pathways, improving the electron migration rate within electrode materials, thus enhancing the charge transfer efficiency in batteries or capacitors [62]. Metal doping is also more suitable for high-temperature environments, improving the stability of batteries under high-temperature conditions. Similar to non-metal doping, it can also enhance the material’s cyclic stability, increase structural strength, and improve energy storage performance. Next, we will introduce some practical examples of metal doping.

In a recent study, Chen and co-authors fabricated electrodes based on SnSe, both with and without Cu doping, employing a straightforward hydrothermal synthesis technique [63]. After optimization, the electrode doped with 10 wt.% Cu showcased an exceptional rate capability, delivering a capacity of 330 mA h g^−1^ at a rate of 20 A g^−1^. Remarkably, it maintained a stable cycling capacity of 304 mA h g^−1^ even after 1000 cycles at a current density of 5 A g^−1^. In another investigation, SnSe confined within graphene showcased a specific capacity of 590 mA h g^−1^ at a rate of 0.05 A g^−1^ and 320 mA h g^−1^ at a high current density of 10 A g^−1^ [64]. In order to enhance the performance by reducing volume variations and aggregation during cycling, Liang and colleagues devised a flower-like structure composed of Mn-doped ZnSe, which offers adjustable electronic properties and improved kinetics for lithium-ion transport [65]. Atomic-level structural engineering alleviates potassium-induced lattice strain and enhances the structural stability of negative electrode materials. Flower-like structures composed of ultra-thin Mn-doped ZnSe nanosheets demonstrated a notable capacity of 345 mA h g^−1^, retaining 95.4% of its capacity after 100 cycles at a low current density of 0.2 A h g^−1^. Furthermore, even after 1000 cycles at 2 A h g^−1^, it maintained a capacity of 167 mA h g^−1^. For instance, Li et al. synthesized wrinkled graphene/cobalt selenide (rGO/CoSe_2_) composite materials, where CoSe_2_ nanoparticles were tightly anchored/encapsulated onto rGO sheets [66]. Compared to pure CoSe_2_, the rGO/CoSe_2_ composite material showed improved cycling and electrochemical performance. This study introduced a novel composite comprising CoSe_2_ and rGO, which serves to mitigate structural degradation resulting from volume expansion while simultaneously enhancing the conductivity of the entire electrode. Consequently, this innovative approach led to a notable enhancement in lithium storage performance. However, Chen et al. synthesized porous copper-doped CoSe_2_ using ZIF-67 as a precursor and selenium powder, where the nanoparticles were interconnected through annealing [67]. This porous material exhibited potential as a negative electrode material for LIBs, adept at accommodating volume expansion. Moreover, its porous structure significantly reduced ion and electron transport pathways, thereby imparting a superior rate performance and ensuring good cycling stability.

**Figure 5:**
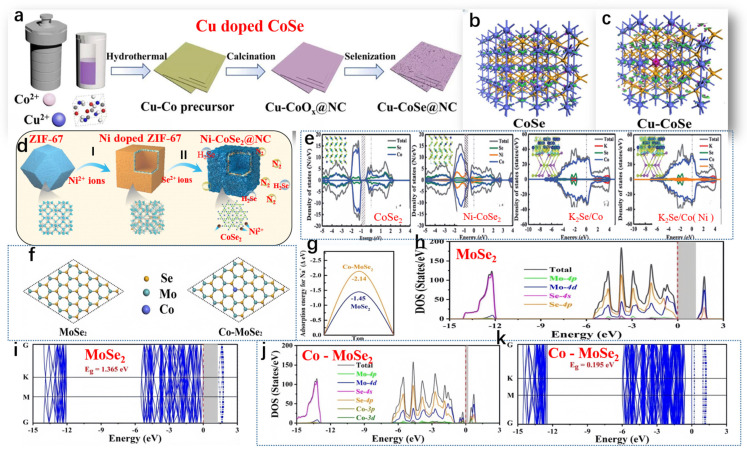
(**a**) Schematic of the Cu-CoSe@NC synthesis process, (**b**) 3 × 3 × 2 CoSe supercell, and (**c**) optimized structure of Cu-doped CoSe. (**a**–**c**) Reproduced from [68] Copyright 2009, Nanoscale. (**d**) Schematic representation of Ni-CoSe_2_@NC preparation and (**e**) density of states (DOS) analysis of CoSe_2_, Ni-CoSe_2_, K_2_Se/Co, and K_2_Se/Co (Ni) heterointerfaces, with insets showing the corresponding charge density differences. (**d**,**e**) Reproduced from [69] Copyright 2022, John Wiley and Sons. (**f**) Optimized structures of MoSe_2_ and Co-MoSe_2_, (**g**) Na^+^ adsorption energies on MoSe_2_ and Co-MoSe_2_, and (**h**–**k**) density of states (DOS) plots for single-layer MoSe_2_ and Co-MoSe_2_. (**f**–**k**) Reproduced from [70] Copyright 2014, Inorganic Chemistry Frontiers.

Geng et al. developed Cu-CoSe@NC, and Figure 5a illustrates the synthetic process of Cu-CoSe@NC [68]. The electrical conductivity of Cu-CoSe@NC and CoSe@NC was analyzed through theoretical calculations. The iso-surface, representing the electron density difference and charge distribution of CoSe, suggests that Co atoms harbor localized electrons and holes (refer to Figure 5b). Analysis of the charge population indicates that Co atoms exhibited a positive charge of +0.36 eV, while Se atoms possessed a negative charge of −0.36 eV. Upon doping, Cu atoms accumulated additional positive charges, reaching +0.39 eV (see Figure 5c). Furthermore, relative to the pristine CoSe, there was an observed increase in the delocalization of electron density difference on iso-surfaces. By comparing the electron density difference and charge distribution between CoSe and Cu-doped CoSe, it becomes evident that the introduction of Cu led to enhanced charge delocalization within CoSe. Hui et al., as shown in Figure 5d, outlined the synthesis of Ni-CoSe_2_@NC in three stages [69]. In brief, they first prepared a zeolitic imidazolate framework (ZIF-67) with a rhombic dodecahedral structure containing Co using a typical coprecipitation method. Significantly, the electrochemical characteristics of the intermediate heterointerfaces in the doped material (0.33 eV) were found to surpass those of bimetallic TMS (0.50 eV), underscoring the importance of meticulous and adjustable doping techniques. Furthermore, a comparison of the density of states (DOS) between bulk CoSe_2_ and Ni-CoSe_2_ was conducted. In the range of ≈−0.5~1.0 eV, the electron states of Ni-CoSe_2_ were higher than those of CoSe_2_, validating the enhancement of conductivity of the nanocomposite material after doping strategies (Figure 5e). Following the conversion reaction, naturally grown K_2_Se/Co and K_2_Se/Co (Ni 10%) heterointerfaces exhibited metallic characteristics, as depicted in Figure 5e. The incorporation of Ni atoms reshaped the heterointerfaces’ electron structure of the electrode material, indicating higher conductivity in K_2_Se/Co (Ni 10%). Zhang et al. depicted the atomic structure, band structure, and projected density of states (PDOS) of MoSe_2_ (2H phase) and Co-MoSe_2_, obtained through DFT calculations, as shown in Figure 5f,g [70]. DFT analysis revealed that MoSe_2_ is a semiconductor with a bandgap of 1.365 eV (Figure 5h–i). Upon Co doping, the bandgap decreased significantly to only 0.195 eV, attributed to the intermediate bandgap states induced by Co doping, as depicted in Figure 5j-k. The PDOS of Co-MoSe_2_ clearly indicated that the 3d orbitals of Co participated in forming small split peaks in both the conduction and valence bands, drastically reducing the bandgap of MoSe_2_ and enhancing conductivity. To observe the effect of Co doping on Na ion adsorption, Na ions were placed at different adsorption sites on MoSe_2_ and CoMoSe_2_. The results indicate that in intrinsic MoSe_2_, the vacancy (Eads = −3.05 eV) was more favorable for Na^+^ adsorption than the Mo top site (Eads = −1.45 eV). After Co doping, the Eads for Na^+^ at both positions significantly decreased. At the Co-doped top site, Eads was −3.82 eV, and around the vacancy near the Co atom, Eads was −2.14 eV. DFT analysis suggested that Co doping not only enhanced the conductivity of MoSe_2_ but also improved the adsorption strength of Na^+^.

**Figure 6:**
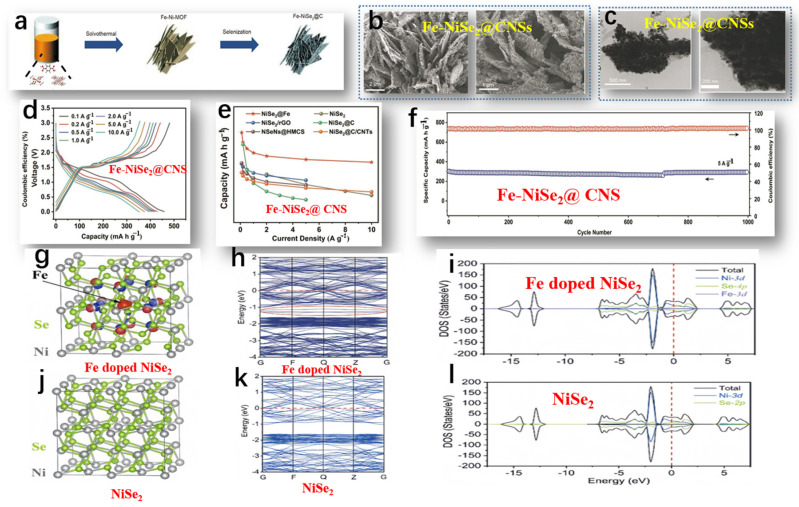
(**a**) Schematic diagram of the preparation process of Fe-NiSe_2_@CNSs. (**b**) SEM image of the morphology of Fe-NiSe_2_@CNSs. (**c**) TEM image of the morphology of Fe-NiSe_2_@CNSs. (**d**) Charge–discharge curves from 0.1 to 10 A g^−1^. (**e**) Rate performance comparison of Fe-NiSe_2_@CNS electrode with reported NiSe_2_-based negative electrodes. (**f**) The long-term cycling performance of Fe-NiSe_2_@CNSs at 5 A g^−1^. (**g**) Optimized charge density difference iso-surface of Fe-doped NiSe_2_. The red iso-surface (iso-value: −0.02×10^−0.3^) indicates the negative charge accumulation region, while the blue iso-surface (iso-value: +0.02×10^−0.3^) indicates the positive charge accumulation region. (**h**) Band structure of Fe-doped NiSe_2_. (**i**) Density of states diagram of Fe-doped NiSe_2_. (**j**) Geometric structure of pure NiSe_2_. (**k**) Band structure of pure NiSe_2_. (**l**) Density of states diagram of pure NiSe_2_. (**a**–**l**) Reproduced from [71] Copyright 2022, Springer Nature.

Liu et al. studied the preparation of Fe-NiSe_2_@CNSs, and the overall preparation process of Fe-NiSe_2_@CNSs is shown in Figure 6a [71]. The morphology of Fe-NiSe_2_@CNSs was observed by SEM and TEM. The SEM image of Fe-NiSe_2_@CNSs (Figure 6b) shows numerous porous 2D NSs with clear boundaries. The average width and thickness were 3 μm and 8 nm, respectively. TEM images confirmed the uniform dispersion of ultra-thin carbon NSs encapsulating nanoparticles with a diameter of 40 nm (Figure 6c). Further confirmation of the excellent sodium storage performance of FeNiSe_2_@CNSs was achieved through rate capability tests (Figure 6d). The Fe-NiSe_2_@CNSs exhibited reversible capacities of 457, 429, 418, 406, 398, 376, and 352 mA h g^−1^ at current densities of 0.1, 0.2, 0.5, 1.0, 2.0, 5.0, and 10.0 A g^−1^, respectively. Upon reducing the current density to 0.1 A g^−1^, the discharge capacity rebounded to 451 mA h g^−1^, underscoring the favorable reversibility of the nanosheets. In contrast, the rate performance of pure NiSe_2_ NS electrodes was not ideal, further illustrating that Fe doping can significantly improve the rate performance and enhance conductivity. The excellent rate performance of Fe-doped NSs exceeded that of other reported NiSe_2_-based composite materials (Figure 6e). In Figure 6f, the enduring cycling behavior of Fe-NiSe_2_@CNSs at 5 A g^−1^ is delineated. Even after 1000 cycles under such conditions, a discharge capacity of 302 mA h g^−1^ was upheld, reflecting a retention rate of 100%. The remarkable electrochemical prowess of Fe-NiSe_2_@CNSs, facilitating Na^+^ transportation, is ascribed to its porous architecture, enhanced conductivity, and fully exposed contact surface. The conductivity of NiSe_2_ was investigated before and after Fe doping via theoretical analysis. Observing the equipotential surfaces of Fe-doped NiSe_2_ (depicted in Figure 6g), it is evident that electrons clustered around the doped Fe atoms, attributed to the lower valence of Fe^3+^ in comparison to Ni^4+^. The equipotential surfaces of pure NiSe_2_ are shown in Figure 6j. Fe doping had no significant effect on the metallic properties and band structure of NiSe_2_. As shown in Figure 6h,k, the conduction bands near the Fermi level in Fe-doped NiSe_2_ were significantly denser compared to those in pure NiSe_2_. Furthermore, Fe doping induced new doping bands within the range of −1 to 1.5 eV in the conduction band (indicated by the red circles in Figure 6g). Density of states (DOS) plots for Fe-doped NiSe_2_ demonstrated better conduction as the valence band crossed the Fermi level (Figure 6i,l). Therefore, the accumulation of more electrons in Fe-doped NiSe_2_ enhanced the conductivity of the battery.

Table 1 mainly presents the performance changes in Fe, Co, Ni, and Mo selenides after heteroatom doping. It can be seen that after a certain number of cycling tests, TMSs showed a significant enhancement in theoretical capacity in SIBs following heteroatom doping. The size and type of metal selenide microspheres, as well as the current density during the cycling process, all had varying degrees of influence on the energy storage performance. Different synthesis methods for anode materials can also have varying impacts on the electrochemical properties of SIBs. For instance, hydrothermal synthesis can produce nanomaterials with high crystallinity and uniform size, making it suitable for constructing complex nanostructures and porous materials. In contrast, the pyrolysis method is suitable for preparing carbon-coated composite materials by thermally decomposing precursor materials. Although solid-state synthesis can yield high-purity crystalline materials, it is challenging to control their morphology and size. This confirms that heteroatom doping is an effective means to improve the energy storage capacity of TMSs in SIBs.

### 3.2. Construction of Heterojunctions

Compared to heteroatom doping, constructing heterojunctions offers higher degrees of freedom and controllability, facilitating the manipulation of bonding modes and interface morphologies between different materials. This aids in optimizing material performance and achieving more precise modulation. The formation of heterojunctions also induces interface effects, leading to adjustments in electronic structure and changes in local material properties, whereas heteroatom doping does not distinctly form interface effects [87,88]. Heterojunction construction can also realize interface engineering, optimizing the interface properties of materials, such as enhancing interface stability, improving interface bonding strength, and reducing defect density, which plays a crucial role in enhancing material stability. Additionally, constructing heterojunctions can improve device performance, such as increasing carrier transport rates, enhancing light absorption capacity, and reducing leakage current, among other benefits [89,90].

Jiao et al. employed a hydrothermal-selenization-coating strategy to prepare heterostructure-decorated SnSe_2_/ZnSe@PDA nano-boxes as negative electrode materials for SIBs [91]. Due to the optimized electrode structure, which mitigated volume expansion during cycling and enhanced sodium-ion adsorption, the material exhibited sustained cycling for 1000 cycles at a current density of 1.0 A g^−1^, along with a high specific capacity of 616 mA h g^−1^. Via a hydrothermal approach, Gao and colleagues successfully synthesized hierarchical nanotubes composed of CoSe_2_-encapsulated MoSe_2_/C [92]. Remarkably, after subjecting the prepared nanotubes to 100 cycles of sodium storage at a current density of 0.1 A g^−1^, they displayed an impressive specific capacity of 450 mA h g^−1^. In a separate investigation, Kang and co-workers synthesized layered MoSe_2_ nanoflowers through a straightforward process involving solvothermal reaction and subsequent annealing [93]. Subsequently, they developed MoSe_2_/MoO_3_ hetero-nanostructured composite materials through post-annealing in an air environment. Impressively, the unoxidized samples showcased a discharge capacity of 347 mA h g^−1^ during the second cycle and maintained 307 mA h g^−1^ after enduring 200 cycles. Conversely, the MoSe_2_/MoO_3_ hetero-composite demonstrated a remarkable specific capacity of 485 mA h g^−1^ after undergoing 200 cycles. Zhang and co-authors incorporated sulfur into freshly synthesized 1T-MoSe_2_ nanospheres to generate composite materials, known as 2H-MoS_2x_Se_2-2x_ [86]. After 100 cycles at a current density of 0.5 A g^−1^, the heterostructure exhibited a reversible discharge capacity of 494 mA h g^−1^, thanks to the incorporation of sulfur heteroatoms, which not only enhanced the cycling stability but also improved the specific capacity, initial Coulombic efficiency, and rate performance. Although the 1T-MoSe_2_ phase boasts a more expandable interlayer structure and superior conductivity, its application in SIBs still necessitates further investigation. In 2017, Zeng, L. introduced heterojunction interface Ni_0.85_Se@MoSe_2_ nanosheet arrays [94]. This heterojunction interface serves a dual purpose: preventing electrolyte aggregation and functioning as a reservoir, thereby promoting swift electrolyte transport. Clear vertical alignment of MoSe_2_ nanosheets was grown on Ni_0.85_Se. Compared to Ni_0.85_Se (401 F g^−1^) and MoSe_2_ (113 F g^−1^), the specific capacitance of the Ni_0.85_Se@MoSe_2_ core-shell electrode (774 F g^−1^) was increased by 2 times and 7 times, respectively. During the same period, the research team also presented NiSe@MoSe_2_ electrodes, showcasing superior cycling stability, with 93.7% retention after 1000 cycles, in contrast to bare NiSe with 72.9% [95]. It is widely recognized that polymers, notably conductive polyaniline (PANI), have often been employed in conjunction with other metals to construct conductive bridges, thereby augmenting electrochemical performance. In a recent study, Sun, X. unveiled a core-shell heterostructure featuring ultrathin nanosheets of Co(OH)_2_/CoSe_2_ as electrode materials for supercapacitor (SC) applications [96]. TEM images confirmed the porous core-shell architecture of Co(OH)_2_/CoSe_2_, underscoring the beneficial closeness between Co(OH)_2_ and CoSe_2_ for energy storage applications. At a current density of 1 A g^−1^ in a 3 M KOH solution, the core-shell electrode demonstrated an impressive specific capacitance of 1283.3 C g^−1^. Moreover, several other core-shell electrode materials have been documented in the literature. It is worth mentioning that the contact between the components of core-shell electrodes is very tight, distinguishing them from other physically mixed composite materials and offering advantages.

**Figure 7:**
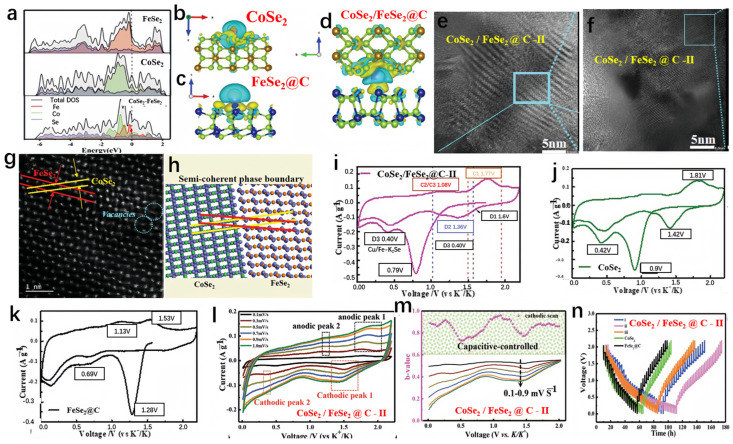
(**a**) The density of states (DOS) analysis of FeSe_2_, CoSe_2_, and CoSe_2_-FeSe_2_. (**b**–**d**) The charge density differences for CoSe_2_, FeSe_2_@C, and CoSe_2_/FeSe_2_@C. (**e**,**f**) HRTEM photos for structural characterization of CoSe_2_/FeSe_2_@C-II. (**g**,**h**) HAADF-STEM images and corresponding schematic diagrams for structural characterization of CoSe_2_/FeSe_2_@C-II. (**i**–**k**) The initial CV curves at a scan rate of 0.1 mV s^−1^ for CoSe_2_/FeSe_2_@C-II (**i**), CoSe_2_ (**j**), and FeSe_2_@C (**k**). (**l**) CV curves for the CoSe_2_/FeSe_2_@C-II electrode at different scan rates. (**m**) The b value for the CoSe_2_/FeSe_2_@C-II electrode was calculated through cathodic scans. (**n**) The voltage curves, obtained through the galvanostatic intermittent titration technique (GITT). (**a**–**n**) Reproduced from [97] Copyright 2021, John Wiley and Sons.

Hui et al. prepared FeSe_2_/CoSe_2_@C, as shown in Figure 7a [97]. The capacity retention of CoSe_2_/FeSe_2_@C after 60 cycles at a current density of 0.05 A g^−1^ was 68 mAh g^−1^. FeSe_2_ exhibited a narrower bandgap, and when the heterojunction structure was achieved, the bandgap shifted near the Fermi level, indicating optimized conductivity compared to bare FeSe_2_ and CoSe_2_. To simplify simulation, bare CoSe_2_ and vacancy-free FeSe_2_ were used to construct the heterojunction interface and investigate charge distribution in the composite material. Figure 7b–d illustrate substantial charge transfer from CoSe_2_ to FeSe_2_, providing additional confirmation of the imbalanced charge distribution evident in the computational findings. Therefore, this study primarily focused on lattice modulation to enhance the diffusion kinetics of vacancy-rich CoSe_2_-FeSe_2_@C in SIBs. Additionally, Figure 7e,f present HRTEM images for structural characterization of CoSe_2_/FeSe_2_@C-II, while Figure 7g,h show HAADF-STEM images and corresponding schematic diagrams for structural characterization of CoSe_2_/FeSe_2_@C-II. In order to highlight the benefits of the heterojunction, the samples obtained were assessed as anodes for SIBs. The initial cyclic voltammetry (CV) test results for CoSe_2_/FeSe_2_@C-II, CoSe_2_, and FeSe_2_@C electrodes at a scan rate of 0.1 mV s^−1^ are shown in Figure 7i–k. The heterojunction anode experienced a notable decline in the reduction peak at 0.79 V, which is ascribed to the development of a solid electrolyte interphase, leading to its disappearance thereafter. Another distinct reduction peak was observed at 0.40 V, consistent with the reaction forming metallic nanoparticles (Co + K_2_Se and Fe + K_2_Se). Conversely, the cathodic peaks at 0.42 V for CoSe_2_ and 0.69 V for FeSe_2_@C were notably elevated compared to that observed for CoSe_2_/FeSe_2_@C. This confirmed the favorable potassium insertion mechanism and excellent electrochemical reversibility of the heterojunction. Potassium ions exhibited a preference for binding with CoSe_2_/FeSe_2_@C, likely attributable to the abundance of vacancies and the presence of controllable semi-coherent phase boundaries. To delve deeper into the energy storage mechanism and kinetics of the electrode, CV tests were performed at various scan rates. Figure 7l shows the curves for CoSe_2_-FeSe_2_@C-II at scan rates of 0.1 to 1.0 mV s^−1^. Additionally, throughout the entire discharge process (Figure 7m), the calculated b value remained greater than 0.75, indicating an almost linear relationship between current and scan rate. To elucidate the specific properties of CoSe_2_-FeSe_2_@C, the galvanostatic intermittent titration technique (GITT) was employed to study its multi-step energy levels (Figure 7n).

**Figure 8:**
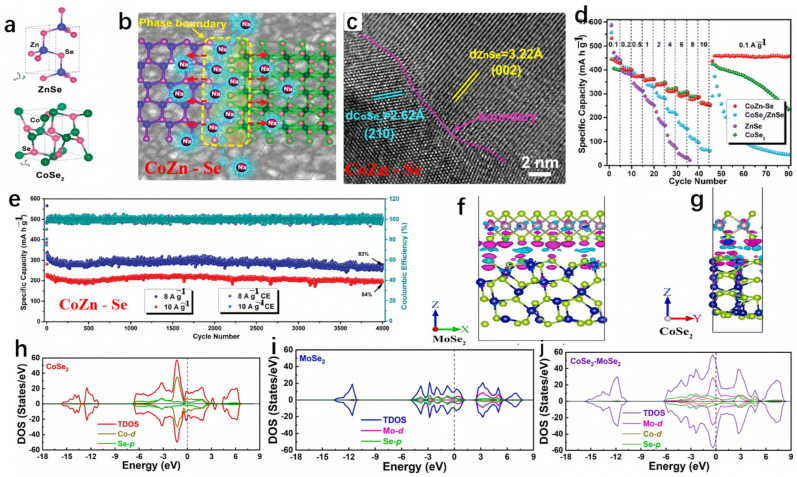
(**a**–**c**) The respective crystal structures, interface effects in CoZn-Se, and corresponding HRTEM images. (**d**) The sodium storage performance of CoZn-Se. (**e**) The rate performance of CoZn-Se compared to ZnSe, CoSe_2_, and CoSe_2_/ZnSe. (**a**–**e**) Reproduced from [98] Copyright 2019, American Chemical Society. (**f**,**g**) The differential charge density of MoSe_2_-CoSe_2_. (**h**–**j**) The density of states (DOS) plots for single-layer CoSe_2_, MoSe_2_ surface, and CoSe_2_-MoSe_2_. (**f**–**j**) Reproduced from [99] Copyright 2021, Elsevier.

Fang et al. devised a bimetallic selenide heterostructure (CoSe_2_/ZnSe nanosheets, referred to as CoZn-Se) and explored the underlying mechanism responsible for its exceptional electrochemical performance [98]. The CoZn-Se can deliver a reversible capacity of 416 mA h g^−1^ at the current density of 0.1 A g^−1^ after 50 cycles. Due to the plentiful lattice distortion-phase boundaries between CoSe_2_ and ZnSe crystallites (illustrated in Figure 8a–c), CoZn-Se demonstrated a reduced Na^+^ adsorption energy in SIBs. This is advantageous, as it favored Na^+^ ion adsorption on the ZnSe side, characterized by high electron density, and facilitated rapid diffusion kinetics. The CoZn-Se heterostructure employed a multi-step redox reaction mechanism, effectively mitigating the stress associated with Na^+^ insertion. As anticipated, CoZn-Se showcased an exceptional high-rate sodium-ion storage capacity when compared to analogous materials, notably maintaining stability through 4000 cycles in a half-cell configuration (as depicted in Figure 8d,e). Zhang et al. conducted theoretical analysis on the conductivity of CoSe_2_ with and without single-layer MoSe_2_ on the surface [99]. As shown in Figure 8f,g, negative charges accumulated on the Se atoms at the interface, while positive charges accumulated in the vacuum chamber between the surfaces. Apparently, the coupling of negative and positive charges in the interface region indicates good conductivity of the heterostructure. Demonstrated by the density of states (DOS) plots in Figure 8h–j, it is evident that both the CoSe_2_ surface and the MoSe_2_-CoSe_2_ heterostructure exhibited conductivity, with the valence band intersecting the Fermi level. Moreover, the addition of MoSe_2_ amplified the conductivity of the CoSe_2_ surface by introducing additional electronic states in both the conduction band (ranging from 3 to 5 eV) and the valence band (spanning from −12 to −15 eV).

Constructing heterojunctions is a promising strategy for enhancing the performance of various electronic and energy storage devices. By integrating different materials with complementary properties, heterojunctions can significantly improve charge separation, carrier mobility, and overall device efficiency. Heterojunctions can enhance the electrochemical performance of battery electrodes by facilitating faster ion transport and reducing charge recombination. They also allow for the customization of the electronic and structural properties of materials, enabling the design of performance-optimized electrodes with improved conductivity, increased surface area, and enhanced structural stability. Developing scalable and cost-effective methods for heterojunction formation is crucial for its commercialization. Ensuring these methods can be implemented at an industrial scale will enable the integration of heterojunction-based materials into commercially viable products. In summary, building heterojunctions holds immense potential for advancing the performance of next-generation energy storage devices. Continued research and development in this field will lead to more efficient, durable, and higher-performing materials.

## 4. Challenges and Recommendations for Future Research Studies

The following is a summary of the challenges faced in the application of TMSs in SIBs and recommendations for future research:

- Further experimental work on SIBs using TMSs as anode materials is crucial, as they are still limited by susceptibility to oxidation or degradation under humid environmental conditions, affecting their performance and lifespan. Additionally, more research is needed to better understand the synthesis and preparation conditions of TMSs and to ensure their operational safety.
- To better understand the application of TMSs as anode materials in SIBs, further molecular dynamics simulations are needed to obtain more valuable molecular interaction data and extract useful information from this data.
- The relationship between the structure and physicochemical properties of electrolytes requires further in-depth investigation to ensure safety across a wide temperature range. Additionally, fundamental parameters, such as the reduction of low-temperature conductivity and the modification of high-temperature structure-driven migration models, also need to be thoroughly studied.
- To gain a deeper understanding of TMSs in SIBs, atomic characterization techniques should be utilized to analyze structural changes, closely observe the microstructure of electrode–electrolyte interfaces, and monitor the crystal structure of TMSs in real time. This approach facilitates the optimization of material-phase stability. Although in situ characterization techniques are essential tools in materials science and engineering, they still face some unavoidable challenges. For example, controlling the environment is difficult, performing precise measurements under high temperature, high pressure, or strong electric fields is challenging, and rapid data changes are hard to capture. The preparation and handling of samples pose significant challenges due to the fragility or sensitivity of the materials involved. Experimental conditions are stringent, and the consumption of time and resources cannot be overlooked. Addressing these issues requires further research investment.
- For a better understanding of the electronic structure of transition metal selenides, appropriate data should be selected to train machine learning models. These trained models can then be applied to new samples of transition metal selenides to predict their electronic structures, thereby providing insights into their properties and characteristics.

Considering these issues, further research is essential to determine the appropriate application of TMSs in SIBs. These studies should focus on the aforementioned aspects.

## 5. Conclusions

Currently, the energy crisis and environmental issues are driving researchers to continuously search for new structures and devices that can replace fossil fuels. Lithium-ion battery technology has matured as a green and effective energy storage device. However, limited lithium salt resources and high prices have been obstacles to its large-scale energy storage. SIBs, on the other hand, have garnered attention due to their abundant natural reserves and similar electrochemical principles compared to LIBs. However, the larger radius of sodium/potassium ions results in greater volume changes and more severe structural damage to negative electrode materials during charging and discharging processes, making the development of suitable negative electrode materials a challenging task.

TMSs, besides being environmentally friendly, having a high theoretical capacity, and being cost-effective, exhibit many unique characteristics compared to TMOs and sulfides. For instance, the nanoarray structure of metal selenides can increase the loading capacity of active materials, and their special layered structure facilitates ion diffusion, resulting in superior cycling stability. Additionally, metal selenides possess inherent metallic properties, superhydrophilicity on the material surface, unique honeycomb arrangement, and robustness of multiple structures. Despite the numerous advantages of TMSs, they still have some shortcomings, such as inadequate conductivity caused by local defects or non-uniformity of metal selenides and volume expansion of the crystal structure during battery charge–discharge cycles due to sodium-ion insertion and extraction.

To address this, many researchers have attempted to combine TMSs with substances such as MXene and rGo. However, the issue of achieving interface consistency and effective bonding between TMSs and MXene or rGo remains unresolved. Additionally, the preparation process of materials formed by the combination of TMSs with MXene or rGo is complex, and it requires high production costs, making large-scale production challenging. To tackle these issues, this paper mainly focused on two aspects: heteroatom doping and heterojunction construction. Heteroatom doping is a simple and effective method that involves introducing defects and altering crystal structures to modify the electronic structure and state of selenides, thereby enhancing their conductivity and improving the material’s rate performance. This paper categorized heteroatom doping into non-metallic and metallic element doping. Non-metallic doping can enhance reaction kinetics, allowing electrode materials to be used in high-power scenarios, thereby improving the overall performance of batteries. On the other hand, metallic element doping introduces additional electron conduction channels, further enhancing the material’s conductivity. Moreover, its stability at high temperatures makes it suitable for high-temperature applications. In contrast to heteroatom doping, constructing heterojunctions offers higher degrees of freedom and allows for more convenient control over the bonding modes and interface morphology between different materials. This approach can enhance the performance and stability of materials, as well as improve device performance by increasing efficiency and reducing power consumption. The formation of heterojunctions can also yield novel functionalities, such as quantum dot effects, which hold promising prospects for future research.

Innovations regarding TMSs in SIBs have also been consistently reported recently, such as Cu_2−x_Se@3D-CN [100], 3DOM-MnFeSe_x_@C [101], G-MoSe_2_/NHPCB [102], and FeSe_2_ [103], including the (CoNi)Se_2_/NC [104], CoSe_2_@NC [105], and so on. It has indeed attracted relevant researchers to study its SIBs performance. While there are some reports on improving the rate performance of TMs in SIBs through doping to enhance their electronic structure, there are still few reports on the combined modulation of TMSs’ sodium-ion adsorption barrier by both element doping and interface effects to jointly regulate the SIBs performance of TMSs. Second, providing in-depth mechanistic insights into the sodium storage behavior of TMS-based anodes is necessary. Understanding the underlying reaction mechanisms and kinetics could provide valuable information for the design and optimization of future anode materials for SIBs. Additionally, the novelty can also stem from the practical applications of TMSs. The assembled full cell to achieve a high energy density can indicate the enormous potential of the TMSs in SIBs energy storage applications. Incremental improvements in these key parameters can have a profound impact on the practical viability of SIBs, which are vital for energy storage solutions.

## Figures and Tables

**Table 1:** Classification summary of heteroatom-doped atoms (PC: propylene carbonate; FEC: fluoroethylene carbonate; DEGDME: diethyleneglycol dimethylether; EC: ethylene carbonate; DEC: diethyl carbonate; DMC: dimethyl carbonate; DME: dimethoxyethane; DMM: dimethylether; PC: propylene carbonate).

Type of Materials	Current Density (A g^−1^)	Cycle No.	Specific Capacity (mA h g^−1^)	Theoretical Capacity (mA h g^−1^)	Electrolytes	Method of Synthesis	Number
**MoSe_2_ microspheres** **(1–2 mm)**	0.2 A g^−1^	50	433 mA h g^−1^	422.6 mA h g^−1^	\	Pyrolytic selenization (300 °C, 3 h)	[72]
**MoSe_2_ microspheres** **(90 nm)**	2.0 A g^−1^	120	552 mA h g^−1^	422.6 mA h g^−1^	1 M NaClO_4_ in PC with 5% FEC additive	Hydrothermal method (180 °C, 12 h)	[73]
**Carbon-coated CoSe_2_**	1.0 A g^−1^	5000	600 mA h g^−1^	494.5 mA h g^−1^	1 M NaCF_3_SO_3_ in DEGDME	Pyrolysis method (500 °C, 3 h)	[74]
**FeCo_2_Se_4_**	0.35 A g^−1^	5000	350 mA h g^−1^	613.3 mA h g^−1^	\	Pyrolytic selenization (300 °C, 1.5 h)	[75]
**MoSe_2_-C**	3.0 A g^−1^	1000	378 mA h g^−1^	422.6 mA h g^−1^	1 M NaClO_4_ in EC/DEC (1:1 vol%) with 5 wt% FEC	Hydrothermal method (200 °C, 12 h)	[55]
**MoSe_2_/N, P-rGO**	0.5 A g^−1^	1000	378 mA h g^−1^	422.6 mA h g^−1^	\	Hydrothermal method (200 °C, 10 h)	[56]
**FeSe_2_ microspheres**	1.0 A g^−1^	2000	372 mA h g^−1^	501 mA h g^−1^	1 M NaCF_3_SO_3_ in DEGDME	Hydrothermal method (180 °C, 12 h)	[76]
**Carbon nanorod-encapsulated Fe_7_Se_8_**	3.0 A g^−1^	500	218 mA h g^−1^	419.3 mA h g^−1^	\	Pyrolysis method (40–750 °C)	[77]
**FeSe_2_/N-C**	10 A g^−1^	10,000	308 mA h g^−1^	501 mA h g^−1^	NaCF_3_SO_3_/DEGDME	Pyrolytic selenization (500 °C, 2 h)	[49]
**NiSe_2_ nanofibers**	0.2 A g^−1^	100	35 mA h g^−1^	494.8 mA h g^−1^	1 M NaClO_4_ (Aldrich) dissolved in a mixture of EC/DMC	Pyrolysis method (450 °C, 3 h)	[31]
**NiSe_2_–rGO-C**	0.2 A g^−1^	100	468 mA h g^−1^	494.8 mA h g^−1^	1 M NaClO_4_ (Aldrich) dissolved in a mixture of EC/DMC	Pyrolytic selenization (300 °C, 10 h)	[31]
**NiSe_2_/rGO**	1.0 A g^−1^	1000	346 mA h g^−1^	494.8 mA h g^−1^	1 M NaCF_3_SO_3_ dissolved in DMC	Hydrothermal method (200 °C, 18 h)	[78]
**Fe-NiSe_2_@CNSs**	1.0 A g^−1^	100	406 mA h g^−1^	494.8 mA h g^−1^	1 M NaPF_6_ in 1,2-DME	Hydrothermal method (150 °C, 3 h)	[71]
**NiSe_2_ microspheres**	10.0 A g^−1^	3000	374 mA h g^−1^	494.8 mA h g^−1^	1 M NaCF_3_SO_3_	Hydrothermal method (120 °C, 10 h)	[79]
**NiSe@C**	3.0 A g^−1^	2000	160 mA h g^−1^	494.8 mA h g^−1^	\	Pyrolysis method (40–500 °C)	[77]
**CoSe_2_–MoSe_2_**	10 A g^−1^	1000	466 mA h g^−1^	494.5 mA h g^−1^	1 M C6H18KNSi2 imide in DEC/EC (*v*/*v*, 1:1)	Pyrolysis method (500 °C, 8 h)	[80]
**O-FeSe_2_ NSs**	1.0 A g^−1^	700	268 mA h g^−1^	501 mA h g^−1^	1 M NaClO_4_ (PC + 5% FEC)	Hydrothermal method (80 °C, 5 h)	[61]
**P-MoSe_2_@rGO**	10 A g^−1^	1450	338 mA h g^−1^	422.6 mA h g^−1^	1 M NaPF_6_ (dissolved in DME = 100 vol%)	Hydrothermal method (200 °C, 12 h)	[53]
**CoSe-SC@NC**	0.2 A g^−1^	100	505.4 mA h g^−1^	388.7 mA h g^−1^	\	Pyrolysis method (800 °C)	[81]
**Cu-CoSe@NC**	5 A g^−1^	800	428.5 mA h g^−1^	388.7 mA h g^−1^	A DMM solution containing 1 M NaPF_6_	Hydrothermal method (550 °C, 3 h)	[68]
**Ni-CoSe_2_@NC-II**	0.1 A g^−1^	100	400.7 mA h g^−1^	494.5 mA h g^−1^	\	Hydrothermal method (450 °C, 2 h)	[69]
**CoSe_2_@CNF**	0.2 A g^−1^	300	1405 mA h g^−1^	494.5 mA h g^−1^	1 M LiPF_6_ in EC/DMC (1:1 in volume)	Pyrolysis method (300 °C, 2 h)	[82]
**Fe_7_Se_8_@C**	3 A g^−1^	500	218 mA h g^−1^	419.3 mA h g^−1^	\	Pyrolysis method (40–750 °C)	[77]
**N-MoSe_2_-based K^+^**	0.2 A g^−1^	30	314 mA h g^−1^	422.6 mA h g^−1^	0.8 M KPF_6_ in EC/DEC (1:1 in volume)	Hydrothermal method (200 °C, 10 h)	[83]
**MoSe_2_/C core/shell**	1 A g^−1^	1000	226 mA h g^−1^	422.6 mA h g^−1^	\	Pyrolysis method (550 °C, 2 h)	[84]
**MoSe_2_@N-HCS**	2 A g^−1^	16,700	158.3 mA h g^−1^	422.6 mA h g^−1^	1 M NaPF_6_ in DIGYME = 100 vol%	Pyrolysis method (600 °C, 6 h)	[85]
**Fe_2_CoSe_4_**	1 A g^−1^	100	615 mA h g^−1^	827 mA h g^−1^	\	Pyrolytic selenization (400 °C, 1.5 h)	[75]
**MoSe_2_@HCNS**	3 A g^−1^	1000	471 mA h g^−1^	422.6 mA h g^−1^	\	Hydrothermal method (500 °C, 5h)	[54]
**2H-MoS_2x_Se_2-2x_**	0.5 A g^−1^	100	494 mA h g^−1^	670 mA h g^−1^	1 M NaClO_4_ in PC with 5% FEC	Pyrolytic selenization (600 °C, 1 h)	[86]
**Fe-NiSe_2_@CNSs**	5 A g^−1^	1000	302 mA h g^−1^	494.8 mA h g^−1^	1 M NaPF_6_ in 1,2-DME	Hydrothermal method (150 °C, 3 h)	[71]
**Co-MoSe_2_@CN**	10 A g^−1^	1000	373 mA h g^−1^	422.6 mA h g^−1^	1,2- DME and 1 M NaPF_6_	Hydrothermal method (70 °C, 2 h)	[70]

## Data Availability

Data are available upon request.
